# Enhanced Adsorption Performance of Oxytetracycline by Desugared Reed Residues

**DOI:** 10.3390/ijerph15102229

**Published:** 2018-10-11

**Authors:** Min Zhou, Tao Zhu, Xiaohua Fei

**Affiliations:** 1 School of Environmental Science and Engineering, Ocean University of China, Qingdao 266100, China; Y2538ycy@126.com; 2 Henan College of Transportation, Zhengzhou 450008, China; cyy18291960210@126.com; 3 Key Laboratory of Subsurface Hydrology and Ecology in Arid Areas, Ministry of Education, Chang’an University, Xi’an 710054, China

**Keywords:** oxytetracycline, reed residues, adsorption, desugarization

## Abstract

The performance of oxytetracycline adsorption by untreated reed roots, stems and leaves, as well as the desugared reed roots, stems and leaves, was investigated with scanning electron microscopy, Fourier-transform infrared spectroscopy, elemental analysis and surface area analysis to understand the adsorption mechanism. The results showed that the adsorption capacities of untreated reed were 416.35 mg/kg for roots, 341.92 mg/kg for stems and 280.21 mg/kg for leaves, and can be increased significantly by a factor of 8–12 after desugarization. The pseudo-first-order kinetic model was more suitable for describing the adsorption kinetics of reed residues, and the isothermal adsorption process was fitted well by both the Langmuir and Freundlich models. The thermodynamic process suggested that the adsorption was a spontaneous endothermic reaction, and mainly physical adsorption-dominated. The desugared reed tissues had a larger surface area and smaller pore area, and the aromaticity of reed residues increased; on the other hand, the polarity and hydrophilicity decreased after desugarization, thus revealing the mechanism of enhanced OTC(oxytetracycline) adsorption by desugared reed residues. This study suggests that the reed residues contribute the complex adsorption ability for both inorganic and organic contaminates. Corruption of the reed can enhance the adsorption; thus, protecting the natural reed residue and letting it naturally corrupt, rather than artificially cleaning it up, can effectively promote the adsorption of pollutants in the environment and protect environmental and public health.

## 1. Introduction

Antibiotics are now widely existing in the environment and have aroused a lot of concern about their numerous dosages, toxicity, and disturbance to the ecosystem’s function [1,2,3]. It has been reported that about 54,000 tons of antibiotics were used in China in 2013, and about 75% of them were applied to animal husbandry, where because of their poorly metabolized features, a 25–75% proportion of the ingested antibiotics are excreted out of the body via urine and feces to enter into soil, sediment and water bodies [2,4]. The residual antibiotics in the environment have promoted the acquisition or independent evolution of highly specific resistance elements in the absence of innate antibiotic production (such as vancomycin resistance in *Streptomyces coelicolor*, *Paenibacillus* and *Rhodococcus* [5]), and the huge number of resistant genes arise through the occurrence of mutation, recombination, co-option, and/or horizontal gene transfer between bacterial strains [6,7]. A recent study of antibiotic resistance genes in the groundwater environment reported that relative abundances of resistance genes range from 6.61 × 10^−7^ to 2.30 × 10^−1^ copies/16SrRNA gene copies [8]. Thus, the study of environmental behavior of antibiotics to achieve risk prediction and management is of great significance. The probable adverse outcomes related to resistance genes may be clinical (death or treatment failure) or economic (costs of care, length of stay), and reflect both treatment delays and the failure of antibiotic treatment to cure infections [9]. Most importantly, the antibiotic-resistant infections can lead to increased death rates [10]. An estimated 700,000 deaths are caused annually by antibiotic-resistant infections worldwide, and 25,000 of those deaths are in Europe. Currently, it is estimated that 10 million people worldwide would be dying annually by 2050 if the problem is neglected [11]. Therefore, finding an effective way to reduce antibiotics in the environment or exposure to humans to choke the production of antibiotic resistance genes is critical for the implementation of environmental and public health.

Oxytetracycline is one of the highest-detected antibiotics in the environment around the world. Chen et al. (2015) detected high concentrations of TCs (Tetracyclines) in the South Gulf region in China, and they found that oxytetracycline may pose a high risk to aquatic organisms [12]. In Turkey, Karcı (2009) [13] reported a maximum OTC concentration of 500 µg/kg in soil, while 1691 µg/kg in Britain was reported by Kay (2004) [14]. A national reconnaissance of 139 streams in the USA showed that the maximum detected concentration of oxytetracycline was 340 ng/L [15]. A study that monitored the veterinary medicines in water bodies in the UK reported that OTC is present at maximum concentrations of 4490 ng/L in the surface water environment [16]. Thus, the residues of OTC in the environment have been confirmed to be a global issue. However, the majority of waste-water treatment plants cannot reduce the residual OTC in waste water for the lack of efficient processes at present. In general, the methods to remove the antibiotics are adsorption [17,18], strong oxidation [19], photodegradation [20], electrochemical treatments [21], membrane filtration [22] and biodegradation. Adsorption can effectively remove OTC with a low concentration in water, and it has been known that OTC can be absorbed by different minerals [23,24], synthetic materials [25,26,27,28], and so on. Plant residues are known as a kind of abundant, cheap and renewable resource, as well as a common biological adsorbent, and have been widely discussed for the effective removal of heavy metals, such as Ni(II) [29] Cd(II) [30], Pb(II) [31] and Cr(III)/(VI) [29,30], nonmetallic contaminated ions, such as F^-^ [32,33], and some hydrophobic organic contaminants, such as caffeine [34], dyes [35] and polycyclic aromatic hydrocarbons (PAHs) [36,37,38,39]. Reed residue is also known as an effective adsorbent, and there have been discussions about its removal performance on fluoride; it has been demonstrated that considerable surface area was favorable for adsorption [33]. However, little is known about the roles of reed residues in the removal of hydrophilic organic pollutants, and especially, the adsorption of antibiotics onto reed residues requires further study. Furthermore, once the ideal adsorption behavior is observed, the reed would exhibit and contribute the complex adsorption ability directly in its natural growth environment for both inorganic and organic contaminates.

In this study, the adsorption behavior of oxytetracycline by different reed residues (roots, stems and leaves) collected from the Weihe River, as well as the desugarization process, were investigated to explore the adsorption behavior and the mechanism, in the hope of achieving effective removal of residual antibiotics in the environment to reduce the production of antibiotic resistance genes and improve the implementation of environmental and public health.

## 2. Materials and Methods

### 2.1. Chemical Reagents

Oxytetracycline was purchased from Boston Biomedical Inc. (Cambridge, MA, USA). The molecular formula of OTC is C_22_H_24_N_2_O_9_ with a molecular weight of 460.44. Methanol was purchased from Waters Company (Milford, MA, USA) in high-performance liquid chromatography (HPLC) grade. All other reagents were of analytical grade.

### 2.2. Sample Collection and Pretreatment

A reed material was obtained from the Chanba ecoregion (Xi’an, China, 34°25′14.45″ N, 109°1′1.46″ E). The ecoregion is a warm, temperate, semi-humid continental monsoon climate which is warm and dry, and alternately wet, with four distinct seasons. The annual average temperature is 13.0–13.7 °C. The population is 9.617 million. Untreated root (R-U), stem (S-U) and leaf (L-U) samples were washed with deionized water, dried in the shade, broken into pieces and passed through a 60-mesh screen and then stored within dehumidification tanks in different clean seals. The original sample was acidified by refluxing with a 6 mol/L HCl solution at 100 °C for 6 h [40], then extracted by vacuum filtration and washed with deionized water until neutral to obtain the desugared root (R-D), stem (S-D) and leaf (L-D) samples, which were finally stored in the same manner as the untreated samples described above.

### 2.3. Adsorption Experiment Methods

**Figure 1:**
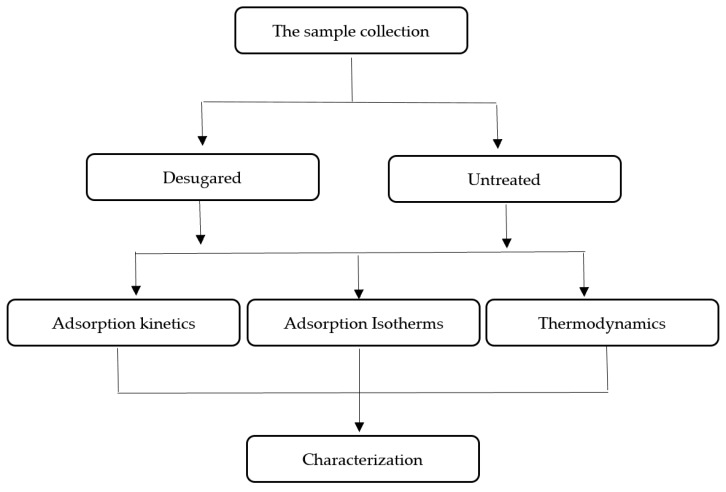
The scheme of the experimental setup.

The initial concentration of OTC used in the kinetic adsorption experiments was 10.0 mg/L at 298 K. In all experiments, the same amount of reed sample was used. The remaining OTC concentration in the solution was determined at regular intervals of 1, 2, 4, 8, 12, 24, 48, 72 and 90 h, and then fitted to the adsorption kinetics model. The isothermal adsorption experiment was carried out at 298 K, and the initial OTC concentration was varied from 5.0 mg/L to 30.0 mg/L. Thermodynamic experiments were carried out at 298 K, 308 K and 318 K, pH = 7.0, and other steps were performed for isothermal adsorption experiments. Fitting and calculation equations were carried out according to the methods described in the literature. The brief experimental setup is described in Figure 1.

### 2.4. Analysis

An ultra-high-performance liquid chromatograph fitted with a Waters TUV detector (Waters UPLC H-Class, ACQUITY UPLC H-CLASS, Waters, MA, USA) and Acquity UPLC BEH C18 (Waters, MA, USA) 1.7 μm 2.1 × 150 mm column was employed for detection and quantification of OTC. Column temperature was 40 °C ± 0.1 °C and sample was stored at 10 °C ± 0.1 °C. Injection volumes of 5 μL, a retention time of 1.530 ± 0.003 min, and a mobile phase of 60% acetonitrile/40% water with a flow rate of 0.1 mL/min were used. OTC was measured at 260 nm. Fourier-transform infrared (FTIR) spectra of untreated and desugared reed residues were recorded by FTIR spectroscopy (model Tensor-27, Bruker, Karlsruhe, Germany) in the 4000–400 cm^−1^ region with a resolution of 4.0 cm^−1^. Elemental (C, H, O, N) analysis of untreated and desugared reed residues was performed using a Vario EL Cube Elemental Analyzer (Elementar, Frankfurt, Germany). The H/C, (N + O)/C and O/C molar ratios of the reed residues were calculated and their aromaticity, polarity and hydrophilicity were evaluated. The surface area and pore area data of the samples followed our previous study: Song et al. (2018) [33].

## 3. Results and Discussion

### 3.1. Adsorption Kinetics

**Figure 2:**
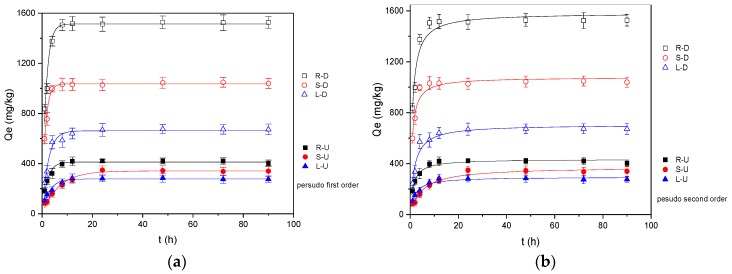
The kinetic model of OTC adsorption by reed residues. (**a**) Pseudo-first-order model; (**b**) pseudo-second-order model.

Under the condition of pH = 7.0, the adsorption of OTC onto reed residues varies with the contact time, as shown in Figure 2. The adsorption process of untreated reed residue was mostly completed within 12 h, and then tended to equilibrium within 24 h, while the adsorption process of desugared reed residue was mostly completed within 12 h and reached equilibrium within 48 h. The desugarization of reed residue can greatly improve the OTC absorption ability. In this study, whether the samples were desugared or not, the adsorption capacity of the roots was the highest, and the adsorption capacity of the untreated reed stems was higher than that of the leaves. Two kinds of kinetics models, pseudo-first-order and pseudo-second-order dynamic models, are used to explain the dynamic results [41,42].

The pseudo-first-order and pseudo-second-order models were employed. The linearized form of the pseudo-first-order kinetic model is given as follows:

(1) $$Q_{t}{=Q}_{e}(1-e^{-K_{1}t}),$$

where Q_e_ (mg/kg) and Q_t_ (mg/kg) are the adsorption of OTC to reed residues at equilibrium and at any time t (h), respectively. K_1_ (1/h) is the rate constant of the pseudo-first-order kinetic model.

The linearized form of the pseudo-second-order kinetic model is given as follows:

(2) $$Q_{t}=\frac{K_{2}Q_{e}^{2}t}{{1+K}_{2}Q_{e}t},$$

where K_2_ (kg/mg·h) is the rate constant of the pseudo-second-order kinetic model. The results showed that all the samples agreed with both the pseudo-first-order and pseudo-second-order kinetic models, but the fitting coefficients (R^2^) of the pseudo-first-order kinetic model were higher, and the calculated Q_e,cal_ values were more consistent with the experimental results (Q_e,exp_) (Table 1). Thus, the pseudo-first-order kinetic model was more suitable for describing the adsorption kinetic data of reed residues. This suggests that the adsorption process was controlled by mono controlling (the sites or the solution concentration only) [18].

### 3.2. Adsorption Isotherms

**Figure 3:**
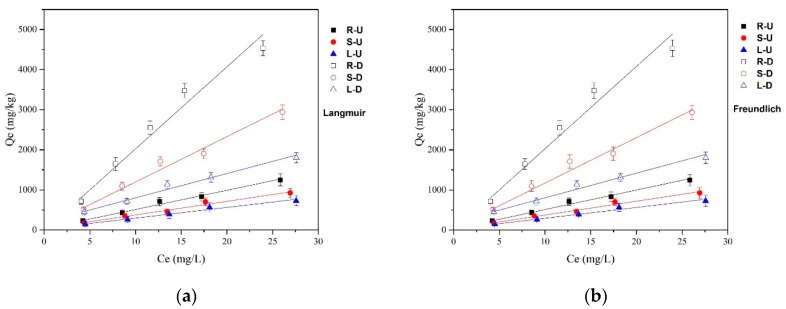
The isothermal models of OTC adsorption by reed residues. (**a**) Langmuir; (**b**) Freundlich.

The isothermal adsorption data of oxytetracycline adsorbed by reed residues were fitted according to the Langmuir model and the Freundlich model [43,44] at 298 K, pH = 7.0, which is shown in Figure 3, and the fitted parameters are listed in Table 2. The high R^2^ values of the Langmuir and Freundlich isotherm models proved that both the Langmuir and Freundlich isotherm models are suitable for describing the behavior of OTC adsorbed by reed residues. K_L_ is the Langmuir adsorption constant associated with the affinity of the binding site, which can represent the adsorption bonding energy [44]. It can be concluded that, after desugarization, the K_L_ values of the reed tissues were all increased to different degrees compared to the untreated samples, indicating that the desugarization process can improve the adsorption intensity or the binding sites to some degree. Similarly, for the Freundlich isotherm model, the values of n are related to the adsorption intensity and are greater than 1 for all reed residues, indicating that the reed residues have good properties to adsorb OTC [45]. In addition, after desugarization, the n values of the reed tissues were all higher than the untreated samples, which also declared that the adsorption capacity was improved because of desugarization.

### 3.3. Thermodynamics

The adsorption of OTC onto reed residues under different temperatures (298 K, 308 K and 318 K, pH = 7.0) was studied and the results are listed in Table 3. The Gibbs free energy change (∆G), the enthalpy change (∆H) and the entropy change (∆S) can be calculated by the following equations:

∆G = −RTlnK_L_, and (3)

∆G = ∆H − T∆S,(4)

where K_L_ (L/mol) is the Langmuir constant, R (8.314 J/mol·K^−1^) is the gas constant, and T (K) is the absolute temperature. Table 3 shows the thermodynamic parameters of the OTC adsorption on the reed residue. The amount of OTC adsorption by reed residues increased with temperature, which indicated that the higher temperatures were beneficial to the adsorption process. The negative value of ΔG indicates the feasibility of OTC adsorption onto the reed residue and the spontaneity of the adsorption process [46]. All of the ∆H values greater than 0 indicated that the adsorption process is endothermic in nature [47]. All of the ∆S values less than 0 suggested an increase in randomness at the solid/liquid interface during the adsorption process [48,49].

From the results observed above, we summarized that the reed residues had a considerable adsorption capacity, especially desugared reed. That means the reed can be employed to remove the residual antibiotics in the environment. The desugared reed had an excellent adsorption behavior, which reminded us that the naturally grown reeds should be allowed to follow their natural growth and life cycles, rather than wasting the labor and material resources to clean them up from the environment. The reeds will better exert their adsorption properties after corruption, remove heavy metals and organic matter from the environment, and control the production of resistance genes to better protect the biosphere and environmental and public health.

### 3.4. Analysis of the Adsorption Mechanism of OTC Adsorption onto Reed Residues

#### 3.4.1. SEM Analysis

**Figure 4:**
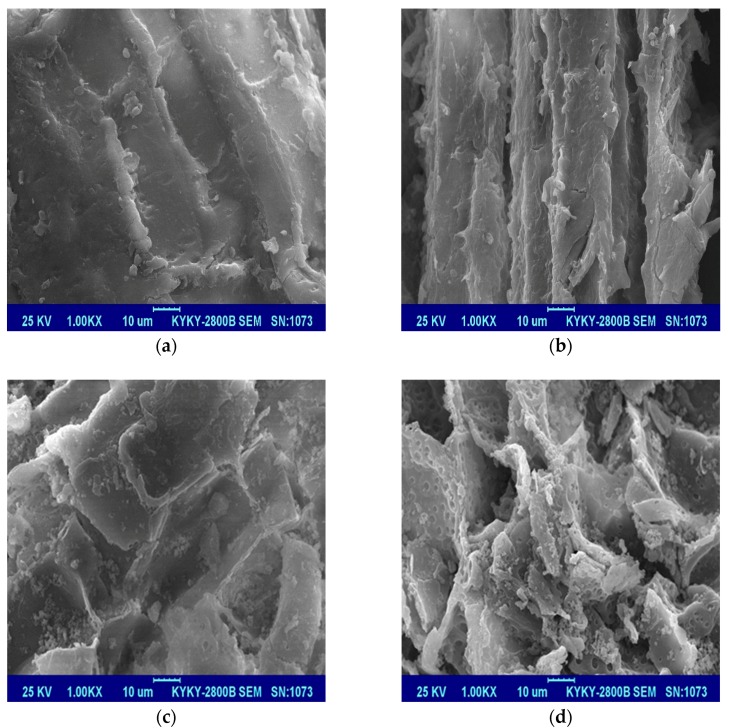
SEM images of the untreated and desugared reed roots before and after OTC adsorption. (**a**) R-U, (**b**) R-U_OTC_, (**c**) R-D, (**d**) R-D_OTC_.

The surface morphologies of the reed residues that were untreated, and the desugared reeds, are exhibited in Figure 4. It can be seen that the R-U surface with abundant morphologies clearly shows the availability of pores and inner surfaces in the SEM image. Moreover, the desugared samples exhibited greater specific surface area and more adsorption sites compared to the untreated reed roots. The skeletal structure was still present in the R-D sample after desugarization, indicating that the surface morphologies of the desugared reed residues were more uneven and rougher compared to the untreated residue.

#### 3.4.2. Elemental Composition Analysis

The elemental characteristics of the reed samples are presented in Table 4. The larger values of (N + O)/C reflect the samples with higher polarity, while larger values of H/C indicate a lower aromaticity. The larger values of O/C reflect a higher hydrophilicity. It can be concluded that the carbon content was increased, while hydrogen and oxygen contents were decreased, after desugarization. More importantly, the polarity indices (N + O)/C of the reed root, stem and leaf decreased after desugarization by a factor of 0.30, 0.16 and 0.21, respectively, the aromaticity indices H/C decreased by a factor of 0.26, 0.15 and 0.14, respectively, and the hydrophilicity indices O/C decreased by a factor of 0.29, 0.15 and 0.18, respectively. This indicated that the polarity and hydrophilicity reduced while the aromaticity increased after desugarization. It proved that the desugarization process can significantly remove the polar tissue, which enhanced the adsorption of OTC. Furthermore, in the process of desugarization, as the aromaticity increased (H/C decreased), the value of logKoc (Koc is the partition coefficient under the standardization of organic carbon) also increased, which corresponded to a significant increase in the adsorption amount of oxytetracycline, indicating that the higher aromaticity was favorable for the OTC adsorbed by reed residues.

#### 3.4.3. Analysis of FTIR

**Figure 5:**
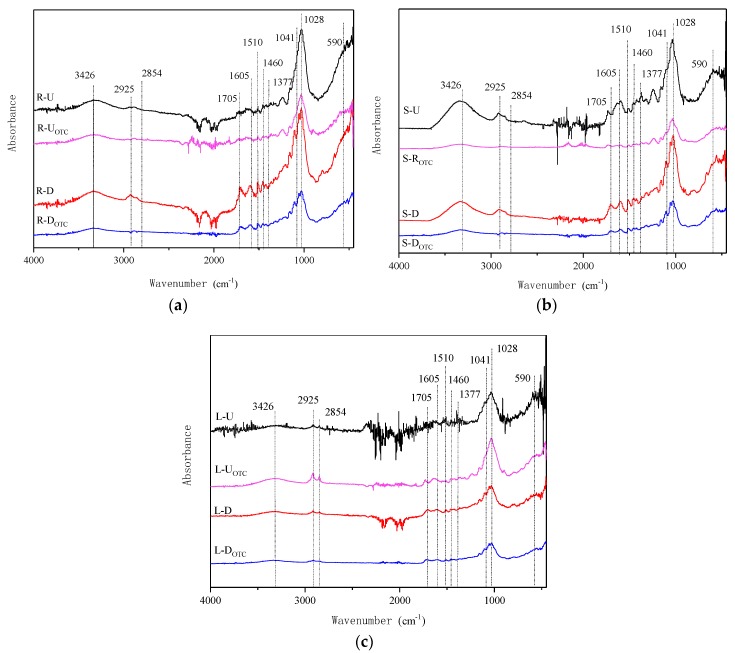
FTIR spectra of the reed (**a**) roots, (**b**) stems and (**c**) leaves.

FTIR spectra of the samples in the range of 4000–400 cm^−1^ before and after desugarization are presented in Figure 5.

For the untreated reed samples, the peaks at 1028 and 1041 cm^−1^ represent the saccharide functional group (C–O–C). After desugarization, the sample mainly absorbed at 1510 cm^−1^ (C=C stretching vibration in lignin), and the functional groups (C=C, C=O and –COOH) at the peaks of 1605 and 1705 cm^−1^ dominated. In addition, the peaks at the C–O–C absorption (1041 and 1028 cm^−1^) assigned to the sugars decreased drastically, while the peaks for the –CH_2_ (2925, 2854 and 1460 cm^−1^) became stronger after desugarization, indicating that the masked fat and aromatic parts were exposed. Therefore, the adsorption amount was greatly increased compared with the untreated sample, and this was in agreement with the kinetic and isothermal experimental results reported above.

#### 3.4.4. Analysis of Surface Area

**Figure 6:**
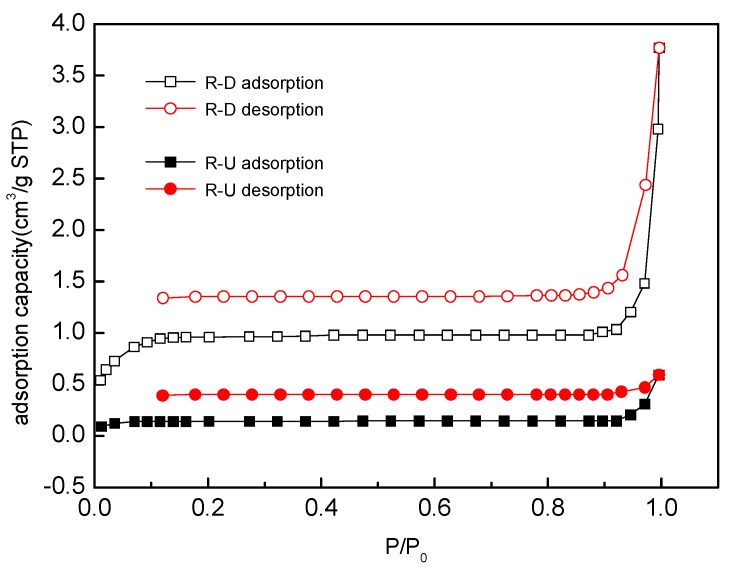
Adsorption–desorption isotherm [33].

The adsorption capacity of OTC came from the reed roots mentioned above. Therefore, the surface area and pore size of untreated and desugared reed root samples were investigated by the adsorption–desorption N_2_ isotherm (Figure 6), and the surface area and pore size data are listed in Table 5 (Song et al. [33]). The adsorption–desorption N_2_ isotherm curve was not a complete circle occurring below 0.1 pressure, indicating that there is irreversible adsorption; that is, the adsorbed N_2_ could not be desorbed, which is due to the micropores of the material having a strong adsorption potential. However, the adsorption amount of N_2_ was almost unchanged with the pressure increases for the untreated root, while it obviously rose for the desugared root. As Table 5 shows, the surface area, pore area and micropore volume of the root of the desugared reed were increased by a factor of 14.27, 1.35 and 1.44, respectively, which further revealed that the adsorption potential of desugared roots was higher than that of untreated ones.

## 4. Conclusions

The following conclusions can be drawn from the current study:

(1) The adsorption amounts of oxytetracycline by untreated reed residues were 416.35 mg/kg for roots, 341.92 mg/kg for stems and 280.21 mg/kg for leaves. The desugarization process can significantly enhance the OTC adsorption by 8–12 times (roots 1525.71 mg/kg, stems 1043.53 mg/kg and leaves 671.94 mg/kg).
(2) The desugared reed tissues had a larger surface area and smaller pore area, and the aromaticity of reed residues increased. On the other hand, the polarity and hydrophilicity decreased after desugarization, which thus revealed the enhanced OTC adsorption mechanism by desugared reed residues. Therefore, protecting the natural reed residue and letting it naturally rot rather than artificially cleaning it up may be effective at promoting the adsorption of pollutants in the environment and protecting environmental and public health.

## Figures and Tables

**Table 1:** Pseudo-first-order and pseudo-second-order model-fitted parameters for OTC adsorption by reed residue.

Samples	Q_e,exp_ (mg/kg)	Pseudo-First-Order				Pseudo-Second-Order			
		R^2^	K_1_ (1/h)	Q_e,cal_ (mg/kg)	RSS/dof	K_2_ (kg/mg·h)	R^2^	Q_e,cal_ (mg/kg)	RSS/dof
R-U	416.35	0.9865	0.5392	416.25	26.50	0.9847	0.0018	439.09	29.84
R-D	1525.71	0.9515	0.6551	1512.88	359.70	0.9514	0.0007	1578.57	360.44
S-U	341.92	0.9855	0.1465	338.83	32.25	0.9849	0.0006	358.22	33.47
S-D	1043.53	0.9767	0.8094	1032.53	75.81	0.9757	0.0012	1085.46	79.00
L-U	280.21	0.9699	0.3671	281.25	29.13	0.9269	0.0017	306.85	39.06
L-D	671.94	0.9421	0.4402	658.52	145.39	0.9053	0.0010	705.78	237.96

**Table 2:** The parameters of isotherm models.

Samples	Q_m_ (mg/kg)	Langmuir			Freundlich		
		K_L_ (L/mg)	R^2^	RSS/dof	1/n	R^2^	RSS/dof
R-U	8238.81	0.0069	0.9897	4717.84	0.91	0.9892	4937.68
R-D	22,468.68	0.0109	0.9782	146,622.09	0.89	0.9701	201,419.04
S-U	5374.83	0.0078	0.9784	5565.49	0.89	0.9768	5984.34
S-D	15,848.97	0.0086	0.9788	52,851.38	0.88	0.9784	53,796.60
L-U	3573.84	0.0095	0.9823	2867.64	0.88	0.9782	3520.16
L-D	5013.84	0.0202	0.9870	10,800.92	0.76	0.9866	11,125.39

**Table 3:** Thermodynamics parameters for OTC adsorption onto reed residues.

Samples	T/K	∆G/kJ·mol^−1^	∆H/kJ·mol^−1^	∆S/J·mol^−1^·K^−1^
R-U	298	−10.36	10.20	69.00
	308	−11.05		
	318	−11.74		
R-D	298	−13.74	4.23	60.30
	308	−14.34		
	318	−14.95		
S-U	298	−9.62	24.25	113.66
	308	−10.76		
	318	−11.89		
S-D	298	−12.53	16.46	97.27
	308	−13.50		
	318	−14.46		
L-U	298	−9.47	46.97	189.38
	308	−11.36		
	318	−13.25		
L-D	298	−11.17	39.62	170.43
	308	−12.87		
	318	−14.58		

**Table 4:** Elemental composition and atomic ratio.

Samples	C (%)	H (%)	O (%)	H/C	(N + O)/C	O/C	Kd (L/kg)	Koc
R-U	42.46	6.06	44.59	1.71	0.81	0.79	51.63	149.95
R-D	51.49	5.46	38.28	1.27	0.57	0.56	204.66	917.52
S-U	44.93	6.12	44.88	1.63	0.76	0.75	37.88	60.43
S-D	50.96	5.91	43.43	1.39	0.64	0.64	120.87	514.95
L-U	42.13	6.10	40.43	1.74	0.76	0.72	29.66	62.95
L-D	50.15	6.26	39.18	1.50	0.60	0.59	80.58	516.13

**Table 5:** The surface area, pore area, micropore volume and pore size of the untreated and desugared roots [33].

Samples	Surface Area (m^2^/g)	Pore Area (m^2^/g)	Micropore Volume (cm^3^/g)	Average Pore Size (nm)
R-U	0.1844	2.2274	0.0009	-
R-D	2.6321	3.0050	0.0013	8.8610
